# Antidiabetic Properties of Curcumin I: Evidence from In Vitro Studies

**DOI:** 10.3390/nu12010118

**Published:** 2020-01-01

**Authors:** Danja J. Den Hartogh, Alessandra Gabriel, Evangelia Tsiani

**Affiliations:** 1 Department of Health Sciences, Brock University, St. Catharines, ON L2S 3A1, Canada; dd11qv@brocku.ca (D.J.D.H.); ag15kh@brocku.ca (A.G.); 2 Centre for Bone and Muscle Health, Brock University, St. Catharines, ON L2S 3A1, Canada

**Keywords:** insulin resistance, diabetes, curcumin, curcuminoids, skeletal muscle, adipose, liver, pancreas

## Abstract

Type 2 diabetes mellitus (T2DM) is a growing metabolic disease characterized by insulin resistance and hyperglycemia. Current preventative and treatment strategies for T2DM and insulin resistance lack in efficacy resulting in the need for new approaches to prevent and manage/treat the disease better. In recent years, epidemiological studies have suggested that diets rich in fruits and vegetables have beneficial health effects including protection against insulin resistance and T2DM. Curcumin, a polyphenol found in turmeric, and curcuminoids have been reported to have antioxidant, anti-inflammatory, hepatoprotective, nephroprotective, neuroprotective, immunomodulatory and antidiabetic properties. The current review (I of II) summarizes the existing in vitro studies examining the antidiabetic effects of curcumin, while a second (II of II) review summarizes evidence from existing in vivo animal studies and clinical trials focusing on curcumin’s antidiabetic properties.

## 1. Introduction

### 1.1. Glucose Homeostasis: Role of Insulin

Insulin is a protein hormone primarily involved in glucose and nutrient homeostasis [1,2,3]. In response to elevated blood glucose levels, β-cells of the pancreatic islets of Langerhans release insulin, which is transported through blood circulation to its target tissues, including skeletal muscle, adipose and liver [1,2,3]. Insulin promotes glucose uptake by skeletal muscle and adipose tissue and suppresses liver’s endogenous production of glucose resulting in restoration of blood glucose back to normal levels [1,2,3].

The mechanism of action of insulin is initiated by binding to its receptor, which is located on the plasma membrane of target cells [1,3,4]. This causes increased receptor tyrosine kinase activity, and subsequent phosphorylation of the insulin receptor substrate (IRS) [1,3,4]. Further downstream, the lipid kinase, phosphatidylinositol-3 kinase (PI3-K) and the serine/threonine kinase Akt/PKB are activated [1,3,4]. In adipose and muscle cells, the glucose transporter (GLUT4) is translocated from an intracellular compartment of the cell to plasma membrane thus increasing the entry of glucose. In liver cells, insulin signaling and action leads to the suppression of glycogenolysis and gluconeogenesis resulting in reduced endogenous glucose production [1].

Impairments in the insulin signaling pathway leads to insulin resistance, defined as reduced responsiveness of target tissues to circulating levels of insulin [3,4,5], and type 2 diabetes mellitus (T2DM) [1,3,4]. Insulin resistance and T2DM are associated with obesity, inflammation, aging and a sedentary lifestyle [3,4,5,6,7,8,9]. Insulin resistance results in hyperglycemia which chronically leads to long-term macrovascular and microvascular damage, and complications such as cardiovascular disease, retinopathy, neuropathy, and nephropathy [3,4,5,6,7,8,9]. Obesity is strongly linked to insulin resistance, with excess plasma free fatty acids (FFAs) impairing the ability of insulin to suppress hepatic glucose output and to stimulate glucose uptake by fat and muscle cells [7,9]. Furthermore, strong evidence has established that chronic inflammation contributes to insulin resistance. Pro-inflammatory cytokines, such as tumor necrosis factor-alpha (TNF-α), reduce the insulin-stimulated insulin receptor and IRS-1 tyrosine phosphorylation, resulting in impaired insulin action and induction of insulin resistance [10].

T2DM accounts for 90%–95% of all diabetes cases, and this global burden continues to rise; diabetes caused 5 million deaths in 2017 in those aged 20 or above, compared to 665,000 deaths in 1990 [11]. It is further estimated that approximately 451 million people, aged 18 or older, live with diabetes worldwide [11]. In addition to the rise in prevalence, diabetes and its related complications exert a significant economic burden on health care systems globally [12,13]. An incidence predictive study on the cost of diabetes in Canada over 10 years has estimated 2.16 million new cases of diabetes which is accompanied by health care costs of $15.36 billion due to acute hospitalizations and prescription medications [14].

Epidemiological studies have suggested that diets high in fruits and vegetables help regulate body weight (obesity) and protect against chronic diseases such as cardiovascular disease, cancer, and diabetes [15,16,17]. However, it is difficult to determine the exact role of individual food components in disease prevention. Specific food components, polyphenols, have continued to gain attention within the scientific community for their potential health benefits and preventive and therapeutic properties against chronic diseases [12,13,18,19,20,21].

Polyphenols have been established to have antioxidant properties [22] and possess a variety of other specific biological effects. Therefore, these components have the potential to prevent diseases through mechanisms that are dependent and independent of their antioxidant properties [23,24,25].

### 1.2. Curcumin

**Figure 1:**
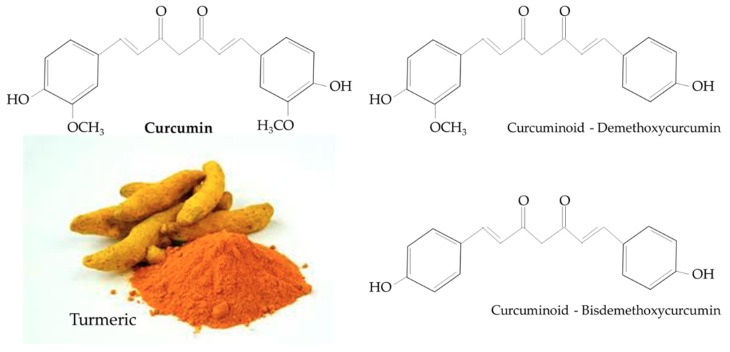
Chemical structure of curcumin and curcuminoids found in turmeric.

Turmeric is a rhizomatous medicinal perennial plant (*Curcuma longa*) that has a rich history of use in Asian countries, including China and South East Asia [26,27]. Curcumin (1,7-bis(4-hydroxy-3-methoxyphenyl)-1,6-heptadiene-3,5-dione), also known as diferuloylmethane, is the main natural polyphenol found in *Curcuma longa* and other *Curcuma* species [27]. Structurally, curcumin includes an enol form of diketone and two methoxylated phenols, the former serving as the active sites that contribute to its antioxidant properties [27]. Curcumin is structurally similar to other curcuminoids, such as demethoxycurcumin and bisdemethoxycurcumin, that differ only with respect to the number of methoxy groups on their aromatic ring (Figure 1) [27]. Turmeric and curcumin have been studied for its potential pharmacological properties such as antioxidant, anti-inflammatory, immunomodulatory, hepatoprotective, nephroprotective, neuroprotective, anti-cancer, anti-atherosclerotic, and antidiabetic properties [28,29,30,31,32,33].

Despite curcumin’s reported anti-inflammatory and antioxidant benefits, it has poor bioavailability, due to its reduced absorption, rapid metabolism and rapid elimination [34]. There are a limited number of studies that examine curcumin’s bioavailability and pharmacokinetics [34,35,36,37,38,39].

In a study by Shoba et al. (1998), the oral administration (2 g/kg body weight (b.w.)) of curcumin to Wistar rats resulted in serum levels of 1.35 ± 0.23 µg/mL (3.66 ± 0.62 µmol/L) after 50 min, while in humans, 2 g of curcumin was administered orally, and resulted in low (0.006 ± 0.005 µg/mL, 0.2 ± 0.1 µmol/L) serum levels within 1 h [34]. However, in another study, curcumin administration of 4–8 g in humans resulted in peak plasma levels of 0.41–1.75 µg/mL (1.11–4.75 µmol/L) after 1 h, indicating that an increased intake of curcumin is required for a better detection in serum [35]. Similarly, curcumin administration (3.6 g/day) in a human clinical trial resulted in plasma curcumin levels of 11.1 nmol/L (0.004 µg/mL) after 1 h [36]. In a study by Sun et al. (2013), intravenous administration, through tail vein, of curcumin (2 mg/kg b.w.) to Wistar rats resulted in serum levels of 6.6 µg/mL (17.92 µmol/L) [37]. In a study by Shoba et al. (1998), healthy human participants were administered with 2000 mg curcumin which resulted in low/undetectable serum levels after 2 h [34]. However, co-administration of 20 mg piperine concomitantly with curcumin showed a 2000% increase in the half-life and bioavailability of curcumin with no adverse effects [34]. These studies [34,35,36,37] indicate that micromolar levels of curcumin can be reached in plasma. Furthermore, the co-oral-administration of curcumin (2000 mg/kg) and piperine (20 mg/kg) to epileptic rats resulted in increased curcumin intestinal absorption and tissue presence [38,39]. Administration of curcumin (500 mg/kg) to rats resulted in peak concentration after 1 h in the intestine (11,830 µg/whole tissue), while blood (490.3 µg/total), kidney (9.03 µg/whole tissue) and liver (135.2 µg/whole tissue) reached peak concentrations after 6 h [39]. The temporal tissue distribution of curcumin was significantly increased when co-administered with piperine, with higher concentrations reached in blood, liver and kidney [39]. More studies should be performed to examine plasma curcumin levels and bioavailability in humans. Overall, these studies suggest that the dosage and route of curcumin administration influences serum levels.

The present review is focused on the antidiabetic effects of curcumin and all existing in vitro studies are presented. The examination/search of the scientific literature focusing on the studies investigating the antidiabetic properties of curcumin revealed an extensive number of original papers and we have organized/summarized all the available information and presented it in two review manuscripts. The first manuscript (Antidiabetic properties of curcumin I: Evidence from in vitro studies) focuses on the in vitro evidence. The second manuscript (Antidiabetic properties of curcumin II: Evidence from in vivo studies) focuses on the in vivo evidence. For the present review, the key words: curcumin, curcuminoid, skeletal muscle, adipocyte, hepatocyte, β-cell, erythrocyte, pancreas and diabetes were searched using the PubMed database. These key words were searched in multiple different combinations to ensure that all existing in vitro studies were included. The studies are presented chronologically, and summary data tables are provided to give the reader easier access to the information.

## 2. Antidiabetic Effects of Curcumin: In Vitro Studies

### 2.1. Effects of Curcumin: In Vitro Adipocyte Studies

In the studies by Kuroda et al. (2005) [40] and Nishiyama et al. (2005) [41], treatment of human adipocytes for 14 days with turmeric ethanol (EtOH) extract (20 µM), containing curcumin, demethoxycurcumin, bisdemethoxycurcumin and ar-turmerone, resulted in significantly increased adipocyte differentiation in a dose-dependent manner, determined by increased glycerol liberation from accumulated triglycerides [40,41]. In addition, human peroxisome proliferator-activated receptor (PPAR)-gamma ligand-binding activity was increased with turmeric EtOH extract treatment (Table 1) [40,41].

Mesenteric adipose tissue was isolated from obese mice and cultured to collect adipose tissue-conditioned medium, used to grow raw 264.7 macrophages [42]. Treatment of raw 264.7 adipose-tissue-conditioned macrophages with curcumin (10 µM) for 24 h resulted in significantly suppressed macrophage migration. Pro-inflammatory mediators tumor necrosis factor-alpha (TNF-α), nitric oxide (NO) and monocyte chemoattractant protein-1 (MCP-1) levels were inhibited with curcumin treatment [42]. In addition, treatment of 3T3-L1 adipocytes with curcumin (10 µM) resulted in significantly reduced MCP-1 levels, similar to the effects shown in macrophages treated with curcumin. These data suggests that curcumin treatment reduces pro-inflammatory cytokine production and macrophage infiltration/activation (Table 1) [42].

Treatment of 3T3-L1 adipocytes with curcumin (20 µM) for 20 min resulted in significantly reduced TNFα-induced IκB degradation and nuclear factor kappa-light-chain-enhancer of activated B cells (NF-κB) nuclear transition, indicating reduced NF-κB activation [43]. TNFα-induced pro-inflammatory cytokine (TNFα, interleukin-1β (IL-1β), and IL-6) and cyclooxygenase-2 (COX-2) messenger ribonucleic acid (mRNA) levels were reduced with 62 h of curcumin treatment. Additionally, curcumin treatment dose-dependently reduced TNFα-induced IL-6 and prostaglandin E_2_ (PGE_2_) secretion, suggesting that curcumin treatment reduces adipocyte inflammatory responses (Table 1) [43].

In a study by Ejaz et al. (2009), treatment of 3T3-L1 adipocytes with curcumin (5–20 µM) for 24 h resulted in suppressed apoptosis, and adipocyte-conditioned medium (ACM)-induced angiogenesis and differentiation from pre- to mature adipocytes (Table 1) [44]. The phosphorylation of 5′ AMP-activated protein kinase (AMPK), acetyl CoA carboxylase (ACC) and carnitine palmitoyltransferase-1 (CPT-1) mRNA levels were increased with curcumin treatment, while glycerol-3-phosphate acyl transferase-1 (GPAT-1) mRNA levels were reduced [44]. Treatment of adipocytes with curcumin increased fatty acid oxidation and reduced fat accumulation [44].

Insulin-stimulated glucose uptake was dose-dependently increased in 3T3-L1 adipocytes treated with curcumin (5–20 µM) for 24 h [45]. In addition, palmitate-induced TNFα and IL-6 mRNA and protein levels were reduced with curcumin treatment. Palmitate-induced nuclear NF-κBp65 protein levels were reduced, indicating that curcumin restricts the translocation of the functionally active subunit p65 to the nuclei. The phosphorylation levels of mitogen-activated protein kinase (MAPK) proteins, c-Jun N-terminal kinase (JNK), extracellular signal-regulated kinase (ERK) and p38, were dose-dependently reduced with curcumin treatment (Table 1) [45].

Treatment of 3T3-L1 adipocytes with curcumin (10–50 µM) for 8 days resulted in reduced adipocyte differentiation [46]. Curcumin treatment significantly increased AMPKα activation/phosphorylation, similar to levels of synthetic AMPK activator, AICAR, while PPAR-γ transcriptional activity was inhibited (Table 1) [46].

In a study by Ahn et al. (2010), treatment of 3T3-L1 adipocytes with curcumin (10–25 µM) for 48 h resulted in suppressed differentiation and reduced the mRNA levels of differentiation-stimulated casein kinase 1α (CK1α), glycogen synthase kinase 3 beta (GSK-3β) and Axin [47]. During differentiation, curcumin treatment inhibited ERK, JNK and p38 phosphorylation and restored β-catenin nuclear translocation dose-dependently [47]. Additionally, mature adipocyte marker (AP2), Wnt direct receptor (Wnt10b and Fz2) and Wnt coreceptor (LRP5) mRNA levels were reduced, while c-master regulator of cell cycle entry and proliferative metabolism (c-Myc) and cyclin D1 mRNA levels were increased with the treatment of curcumin (Table 1) [47].

Treatment of rabbit subcutaneous adipocytes with curcumin (5–20 µM) for 24 h resulted in a dose-dependent increase in cholesterol efflux [48]. In addition, PPARγ, liver X receptor (LXRa) and ATP-binding cassette transporter A1 (ABCA1) mRNA levels were increased with curcumin treatment (Table 1) [48].

In a study by Kim et al. (2011), treatment of 3T3-L1 adipocytes and human primary preadipocytes with curcumin (5–30 µM) for 6 days dose-dependently reduced differentiation, with 80% inhibition shown with 12.5 µM [49]. Curcumin treatment decreased intracellular lipid accumulation and reduced adipocyte marker (CCAAT/enhancer-binding protein (C/EBP)β, C/EBPα, PPARγ, leptin, adiponectin and resistin) mRNA levels. Early adipocyte differentiation was inhibited, mitotic clonal expansion (MCE), cell-cycle entry into S phase, S to G2/M phase transition of confluent cells and cell-cycle regulating proteins (cyclin A and cyclin-dependent kinase 2 (CDK2)) protein levels were reduced (Table 1) [49]. These data suggest that curcumin treatment acts to reduce adipocyte differentiation by the modulation of the MCE process.

Treatment of 3T3-L1 cells with curcumin (20 µM) for 48 h resulted in fast- (reversible non-covalent fast combination with enzyme) and slow-binding (irreversible covalent modification of enzyme) inhibition of fatty acid synthase [50]. This occurred with noncompetitive inhibition in respect to nicotinamide adenine dinucleotide phosphate (NADPH), and partial competitive inhibition against acetyl-CoA and malonyl-CoA with curcumin treatment. In addition, curcumin treatment significantly reduced adipocyte differentiation, PPARγ and cluster of differentiation 36 (CD36) mRNA levels and lipid accumulation, suggesting reduced adipogenesis [50].

In a study by Kim et al. (2011), treatment of 3T3-L1 preadipocytes and adipocytes with curcumin (20 µM) and polyethylene glycol for 48 h resulted in increased retention of curcumin within the cells, particularly within preadipocytes and significantly reduced adipocyte differentiation with no toxic effects. Adipogenic differentiation markers (C/EBPβ, PPARγ, C/EBPα, leptin, adiponectin and resistin) mRNA levels were significantly reduced following 6 days of curcumin and polyethylene glycol treatment (Table 1) [51].

Treatment of 3T3-L1 adipocytes with curcumin (10–20 µM) for 24 h resulted in significantly reduced TNFα- and isoproterenol-induced lipolysis [52]. The phosphorylation of ERK1/2 and perilipin were reduced with curcumin treatment, while total perilipin protein level were increased. In addition, hormone-sensitive lipase translocation from the cytosol to the lipid droplets was significantly reduced with curcumin treatment (Table 1) [52].

Treatment of 3T3-L1 adipocytes with curcumin (10 and 50 µM) and sodium benzoate (10 and 20 µM) for 24 h resulted in a dose-dependent decrease in lipopolysaccharide (LPS)-induced leptin levels [53]. In addition, curcumin treatment decreased LPS-induced IL-6 secretion (Table 1) [53].

Bone marrow mesenchymal stem cells (MSCs) are multipotent cells that have the capacity to differentiate into osteoblasts and adipocytes. Treatment of rat bone marrow MSCs with curcumin (15 µM) for 10 days significantly reduced adipocyte differentiation and inhibited PPARγ2 and C/EBPα mRNA levels (Table 1) [54].

In a study by Hirzel et al. (2013), treatment of human adipocytes with curcumin (15 µM) for 24 h significantly reduced reactive oxygen species (ROS) production measured by the 2′,7′-dichlorohydrofluorescin diacetate assay, while ROS production was unchanged when measured by the nitroblue tetrazolium assay [55]. These data suggest that curcumin oxidizes ROS production.

Treatment of primary rat adipocytes with curcumin (20 µM) for 45 min significantly inhibited basal and insulin-stimulated glucose transport [56]. This inhibition of glucose uptake occurred in cells pre-treated with curcumin and in cells treated with curcumin immediately before glucose transport was measured. In addition, curcumin treatment did not affect the protein level of phospho-protein kinase B/Akt suggesting that these effects on glucose transport is not by the inhibition of insulin signaling or GLUT4 translocation (Table 1) [56].

In a study by Priyanka et al. (2014), treatment of 3T3-L1 adipocytes with curcumin (20 µM) for 24 h significantly protected the cells from toxic hypoxic effects by protecting mitochondria and reducing inflammation [57]. Curcumin treatment reduced hypoxia-induced cell injury, hypoxia-inducible factor 1-alpha (HIF-1α) mRNA level and ROS production. In addition, lactate and glycerol release, lipid peroxidation and protein oxidation were dose-dependently reduced with curcumin treatment [57]. Antioxidant enzymes (superoxide dismutase (SOD) and catalase (CAT)) activities were significantly increased with curcumin, while pro-inflammatory cytokines (TNFα, IL-6, IL-1β and interferon gamma (IFNγ)) secretion were reduced [57]. Mitochondria dysregulation was significantly reduced with curcumin treatment, with increased mitochondrial biogenesis, mitochondrial membrane potential and reduced mitochondrial superoxide production and transition pore permeability (Table 1) [57].

Treatment of 3T3-L1 adipocytes with 10 µM Gambigyeongsinhwan (GGH(4), containing 60% curcumin) or 10 µM curcumin directly, resulted in significant inhibition of lipid accumulation and adipocyte-specific mRNA levels of PPARγ, adipocyte protein 2 (aP2) and C/EBPα [58]. In addition, curcumin or GGH(4) treatment increased mitochondrial (CPT-1, medium-chain acyl-CoA dehydrogenase (MCAD) and very-long-chain acyl-CoA dehydrogenase (VLCAD)) and peroxisomal (acyl-coenzyme A oxidase 1 (ACOX) and thiolase) mRNA levels. PPARα reporter mRNA levels were increased with curcumin or GGH(4) treatment compared to control nontreated cells (Table 1) [58].

SW872 adipocytes treated with curcumin had reduced cell viability, and increased apoptosis (Table 1) [59]. Curcumin treatment increased Bcl-2-associated X (Bax) and decreased B-cell lymphoma 2 (Bcl-2), resulting in an upregulation of the Bax/Bcl-2 ratio. The release of cytochrome c from the mitochondria into the cytosol was increased, as was caspase-dependent poly (ADP) ribose polymerase (PARP) cleavage with curcumin treatment [59].

Treatment of 3T3-L1 adipocytes with curcumin (0–50 µM) for 0–24 h dose- and time-dependently reduced Akt protein levels, while IRS-1 and insulin receptor β (IRβ) protein levels were unchanged [60]. In addition, insulin-stimulated glucose uptake and GLUT4 plasma membrane levels were reduced with curcumin treatment. Autophagy p62 protein levels were reduced, while microtubule-associated protein light chain 3 (LC3)-II protein levels and LC3-II/LC3-I ratio were increased with curcumin treatment [60]. Adipocytes co-treated with either autophagy inhibitors chloroquine (CQ) or bafilomycin A (BFA) and curcumin resulted in restoration of Akt protein levels, suggesting that curcumin reduces Akt protein levels through increased autophagy activities (Table 1) [60].

In a study by Song and Choi (2016), treatment of 3T3-L1 adipocytes with 10 µg/mL turmeric extracts for 24 h resulted in significantly reduced lipid accumulation and leptin levels, while the levels of free fatty acids and glycerol were increased [61]. In addition, turmeric treatment increased adipose triglyceride lipase and hormone-sensitive lipase mRNA levels, suggesting increased triglyceride degradation (Table 1) [61].

Treatment of primary adipocytes with curcumin (20 µM) for 8 days resulted in differential expression of lipid metabolism and mitochondrial biogenesis proteins [62]. Fourteen (acetyl coenzyme A carboxylase A2 (ACCA2), aconitase 2 (ACO2), aldehyde dehydrogenase 2 family member (ALDH2), ATP synthase F1 subunit beta (ATP5B), ATP synthase peripheral stalk subunit D (ATP5H), citrate synthase (CS), dihydrolipoamide dehydrogenase (DLD), glutamate dehydrogenase (GLUD1), hydroxyacyl-CoA dehydrogenase trifunctional multienzyme complex subunit alpha (HADHA), heat shock 60kDa protein 1 (HSPD1), heat shock protein family E (Hsp10) member 1 (HSPE1), malate dehydrogenase 2 (MDH2), peroxiredoxin-5 (PRDX5), and voltage dependent anion channel 1 (VDAC1)) involved in transport or transfer of fatty acids or proteins involved in lipid metabolism were influenced by curcumin treatment. Curcumin treatment significantly reduced ALDH2, ATP5B and PRDX5 expression [62]. ANXA2 and HSP90 protein levels were reduced, while ATP5B, HSL, CA3 and MDH protein levels were increased with curcumin treatment. In addition, thermogenesis genes (uncoupling protein (Ucp)1, Ucp2 and Ucp3) mRNA levels were increased with curcumin treatment (Table 1) [62].

Treatment of 3T3-L1 preadipocytes with curcumin (20 µM) for 72 h resulted in significantly reduced adipocyte differentiation and development of mature adipocytes [63]. Curcumin treatment reduced S-phase kinase-associated protein 2 (Skp2) protein accumulation and induced p27 protein accumulation and G1 arrest. p27 mRNA levels and ubiquitin proteasome activity were reduced with curcumin treatment, suggesting that p27 proteolysis is mediated by the attenuation of Skp2 and proteasome activity [63].

In addition to curcumin, demethoxycurcumin (DMC) and bisdemethoxycurcumin (BDMC) curcuminoids are polyphenols present in turmeric. In a study by Lai et al. (2016), treatment of 3T3-L1 preadipocytes with BDMC (25 µM) for 30 min resulted in significant repression of lipid accumulation and suppressed adipogenesis by reduced MCE and adipocyte differentiation genes (C/EBPα and PPARγ) protein levels (Table 1) [64]. In addition, BDMC treatment induced cell cycle arrest at the G0/G1 phase and reduced cyclin A, cyclin B, p21 and phosphorylation/activation of MAPKs (phospho-ERK, phospho-p38 and phospho-JNK) levels were reduced [64].

Treatment of 3T3-L1 and primary adipocytes with curcumin (20 µM) for 6 days induced browning of white adipocytes and mitochondrial biogenesis [65]. Curcumin treatment increased brown fat markers (PGC-1α, PPARγ, PR domain containing 16 (PRDM16) and Ucp1) protein and PGC-1α mRNA levels. In addition, fat oxidation mitochondrial CPT1 and cytochrome c protein levels were increased with curcumin treatment [65]. Curcumin treatment also increased both phosphorylated and total AMPK. PGC-1α, PRDM16 and Ucp1 protein levels were significantly reduced with the co-treatment of curcumin and AMPK inhibitor dorsomorphin, suggesting that curcumin’s effects are through AMPK activation (Table 1) [65]. This study clearly indicates that curcumin treatment has potential as a treatment against obesity, insulin resistance and T2DM.

In a study by Pan et al. (2017), treatment of 3T3-L1 adipocytes with curcumin (20 µM) for 24 h resulted in significantly improved glucose and lipid metabolism [66]. Curcumin treatment reduced glycerol release, while glucose uptake was increased. In addition, PPARγ and C/EBPα mRNA and protein levels were increased with curcumin treatment (Table 1) [66].

Treatment of 3T3-L1 adipocytes with curcumin (20 µM) for 24 h resulted in reduced hypoxic-induced inflammation and angiogenesis [67]. Curcumin treatment reduced MCP-1 and leptin levels, while adiponectin levels were increased. Resistin and toll-like receptor 4 (TLR-4) mRNA levels, and NF-κB p65 nuclear faction and phosphorylated JNK protein levels were reduced with curcumin treatment, suggesting reduced inflammation. Insulin sensitivity was improved with curcumin treatment, with decreased hypoxic-induced serine phosphorylation of IRS-1 and increased IRS-2 protein levels [67]. In addition, hypoxic-induced glucose uptake and GLUT1 mRNA and protein levels were reduced, while GLUT4 mRNA and protein levels were increased with curcumin treatment. The hypoxic-induced increase in pro-angiogenic factors (MMP-2, MMP-9 and VEGF) and angiopoietin like protein 4 (Angpt4) levels were abolished with curcumin treatment, indicating improve angiogenesis (Table 1) [67].

Treatment of 3T3-L1 adipocytes with curcumin-3,4-dichloro phenyl pyrazole (CDPP) (20 µM) for 7 days resulted in significant inhibition of adipocyte differentiation and decreased lipid accumulation [68]. In addition, CDPP treatment decreased C/EBPα, αP2, SREBP1c and PPARy mRNA and FAS protein levels. Lipid metabolism and fatty acid synthase protein levels were reduced with CDPP treatment [68]. Mitochondrial biogenesis (UCP1 and PGC-1α) protein levels were increased, as well as oxygen consumption rate, suggesting enhanced energy utilization. CDPP treatment increased G1-phase cell cycle arrest and further arrest in S-phase, while cyclin D1, cyclin D3, CDK2, CDK4 and CDK6 protein levels were reduced (Table 1) [68].

Treatment of primary adipocytes and mouse brown adipocyte cells (mBACs) with curcumin (2 µM) for 10 h resulted in improved thermogenesis [69]. Primary adipocytes treated with curcumin have increased UCP1, PPARα and PGC-1α mRNA levels. Curcumin treatment increased UCP1, PPARα, PPARγ and PGC-1α mRNA levels in mBACs (Table 1) [69].

In a study by Wang et al. (2019), treatment of human bone marrow MSCs with curcumin resulted in a dose-dependent reduction in adipocyte differentiation and lipid accumulation [70]. Curcumin treatment inhibited adipogenic PPARγ, C/EBPα, fatty acid binding protein 4 (FABP4) and Kruppel-like factor 15 mRNA and protein levels (Table 1) [70].

Overall, these studies suggest that treatment of adipocytes with curcumin results in reduced adipocyte differentiation, lipid accumulation and inflammation. Curcumin treatment significantly reduces macrophage infiltration and migration, resulting in reduced pro-inflammatory cytokine production. In addition, curcumin treatment suppressed adipogenic (C/EBPβ, C/EBPα, PPARγ, leptin, adiponectin and resistin) and proliferative (MMP1, MMP2, MMP3, SDF1 and VEGF) mRNA and protein expression. Mitochondrial biogenesis and membrane potential were improved, while mitochondrial superoxide production was reduced with curcumin treatment (Table 1).

It should be noted that two studies indicate effects of curcumin against its use in insulin resistance and diabetes. Studies by Green et al. (2014) [56] and Zhang et al. (2016) [60] showed that treatment of adipocytes with curcumin resulted in reduced insulin-stimulated glucose uptake and GLUT4 translocation to the plasma membrane [56,60]. Further research is required to clarify the effects of curcumin on adipose tissue. The majority of studies indicate antidiabetic and anti-lipogenic effects.

### 2.2. Effects of Curcumin: In Vitro Hepatocyte Studies

In a study by Xu et al. (2003), treatment of hepatic stellate cells (HSCs) with curcumin (30 µM) resulted in significantly reduced proliferation and reduced α1(I) collagen, alpha smooth muscle actin (α-SMA) and fibronectin mRNA levels [71]. Curcumin treatment prevented cell cycle-stimulating cyclin D1, D2 and enhanced cell cycle-inhibitory p21 and p27 protein levels. In addition, curcumin treatment induced apoptosis by increased caspase-3 activity and reduced Bcl-2 mRNA levels. Curcumin treatment increased PPAR-γ mRNA and NF-κB and PPAR-γ activity, suggesting reduced transcriptional regulation of oxidative stress and HSC activation (Table 2) [71].

Treatment of isolated hepatocytes with curcumin (25 µM) for 120 min significantly reduced hepatic glycogenolysis and gluconeogenesis from dihydroxyacetone phosphate by 20% and 35%, respectively [72]. The co-treatment of curcumin and insulin further reduced hepatic glycogenolysis and gluconeogenesis, suggesting that this inhibition by curcumin is independent of insulin. In addition, hepatic glucose-6-phosphatase (G6Pase) and phosphoenolpyruvate carboxykinase (PEPCK) activity were reduced, while fructose-1,6-bisphosphatase (FBPase) activity was unaffected with curcumin treatment. The phosphorylation of AMPKα threonine 172 residue was increased with curcumin treatment (Table 2) [72].

Treatment of H4IIE rat hepatoma and Hep3B human hepatoma cells with curcumin (2–50 µM) resulted in significantly reduced dexamethasone-induced PEPCK and G6Pase activity, indicating reduced gluconeogenesis (Table 2) [73]. In addition, curcumin treatment increased AMPK phosphorylation and downstream ACC phosphorylation, suggesting that the gluconeogenesis suppressing may be mediated by AMPK activation [73].

HSCs are the primary effector cells responsible for collagen production during hepatic fibrogenesis and can be activated by insulin to worsen the condition. In a study by Lin et al. (2009), treatment of HSCs with curcumin for 1 h dose-dependently reduced insulin-induced HSC activation (Table 2) [74]. Curcumin treatment reduced pro-mitogenic (platelet-derived growth factor beta receptor (PDGF-βR) and epidermal growth factor receptor (EGFR)) and fibrotic (α-SMA and α-II-collagen) mRNA and protein levels, while cyclin-dependent kinase inhibitors (p21 and p27) and PPARγ mRNA and protein levels were increased. In addition, curcumin treatment promoted HSC apoptosis by reduced Bcl-2 and increased Bax mRNA and protein levels [74]. Curcumin treatment attenuated insulin-induced oxidative stress by reducing intracellular ROS levels and increasing glutathione (GSH) protein, GSH/glutathione disulfide (GSSG) ratio and glutamate cysteine ligase (GCL) activity. The phosphorylation of insulin-signaling proteins (insulin receptor (InsR), ERK1/2, JNK, PI3K and Akt) were significantly reduced with curcumin treatment [74].

Treatment of isolated rat hepatocytes with curcumin resulted in dose discrepancies between 1 µM and 10 µM, as the higher concentration promoted oxidative stress and hepatocytoxicity and the lower concentration protected from lipid peroxidation and cytochrome c translocation [75]. Curcumin treatment (10 µM) significantly increased glutathione depletion, necrosis and apoptosis with increased caspase-3 activity (Table 2) [75].

In a study by Tang and Chen (2010), treatment of rat HSCs and immortalized human hepatocytes with curcumin (20 µM) for 1 h resulted in significantly reduced leptin-induced intracellular glucose levels and GLUT4 membrane protein levels, indicating reduced GLUT4 translocation [76]. In addition, the leptin-induced phosphorylated insulin signaling, IRS-1, PI3K and Akt protein levels were reduced with curcumin treatment. Curcumin treatment significantly increased glucokinase activity and intracellular glucose-6-phosphate levels, resulting in the decreased glucose levels (Table 2) [76]. This data suggests that treatment of HSCs with curcumin protects against leptin-induced HSC activation and hepatic fibrogenesis by reduced intracellular glucose levels and insulin signaling.

Treatment of Huh7 hepatocytes with curcumin (20 µM) for 48 h resulted in increased paraoxonase 1 (PON1) transactivation [77]. In addition PON1 mRNA and protein levels were increased with curcumin treatment, suggesting improved antiatherogenic activity (Table 2) [77].

Pre-treatment of LO2 hepatocytes with curcumin significantly attenuated glucose oxidase-induced oxidative stress and insulin resistance [78]. Curcumin treatment reduced glucose oxidase-induced intracellular ROS production, glutathione depletion and MDA levels, and decreased lactate dehydrogenase (LDH) and aspartate amino transferase (AST) activities [78]. These effects were prevented with nuclear factor erythroid 2–related factor 2 (Nrf2) inhibitor wortmannin, suggesting that curcumin’s effects are mediated by Nrf2 signaling and nuclear translocation [78].

In a study by Kuo et al. (2012), treatment of primary rat hepatocytes with curcumin (10 µM) for 12 h resulted in suppressed high free fatty acid (HFFA)-induced apoptosis and oxidative stress and decreased ROS production and ATP depletion [79]. Curcumin treatment reduced HFFA-induced phosphoenolpyruvate carboxykinase and G6Pase protein levels and increased mitochondrial biogenesis transcription factors (PGC1α, nuclear respiratory factor 1 (NRF1) and mitochondrial transcription factor A (Tfam)) mRNA levels. In addition, curcumin treatment improved cell survival by increased mitochondrial membrane potential (MMP) and reduced NF-κB p65 mRNA (Table 2) [79]. These data suggest that curcumin treatment protects hepatocytes against HFFA-induced lipo-apoptosis and mitochondrial dysfunction by increased mitochondrial biogenesis.

Treatment of AML-12 cells with curcumin (50 µM) for 2 h attenuated the iron overload-induced insulin resistance and improved insulin signaling [80]. Curcumin treatment increased insulin-stimulated Akt phosphorylation in iron overloaded hepatocytes. In addition, iron overloaded-induced ROS and MDA levels, indicators of oxidative stress and lipid peroxidation, respectively, were significantly reduced with curcumin treatment. Curcumin treatment also reduced phosphorylated JNK and p38 protein levels, two pathways involved in stress signaling (Table 2) [80].

Treatment of primary rat hepatocytes with curcumin (15 µM) for 24 h resulted in significantly increased apoB-48 and reduced apoB-100 lipid formation [81]. Curcumin treatment significantly increased apolipoprotein B mRNA editing enzyme catalytic polypeptide 1 (APOBEC-1) mRNA and protein levels, suggesting a shift in lipid metabolism towards apoB-48 containing lipid particles. In addition, curcumin treatment increased the C-to-U editing signal and apoB mRNA editing level, indicating further refinement of the lipid particle metabolism towards apoB-48 (Table 2) [81].

In a study by Canfran-Duque et al. (2014), treatment of HepG2 human hepatocarcinoma cells and THP-1 macrophages with curcumin increased U18666A-induced cholesterol release (Table 2) [82]. U1866A impaired intracellular cholesterol trafficking by sequestering cholesterol within endosome/lysosomes. Treatment with curcumin attenuated these effects. In addition, curcumin treatment increased lysosomal β-hexosaminidase enzyme levels and exosome marker (flotillin-2 and CD63) positive granules, suggesting increased endosome secretion [82]. These data suggest that curcumin treatment promotes exosome secretion.

In a study by Tai et al. (2014), treatment of HepG2 cells with curcumin for 24 h significantly increased low-density lipoprotein (LDL) uptake by increased LDL receptor surface expression and activity [83]. LDL receptor surface expression is negatively post-translationally regulated by proprotein convertase subtilisin/kexin type 9 (PCSK9). Curcumin treatment significantly reduced PCSK9 mRNA and protein levels and promoter activity. In addition, hepatocyte nuclear factor 1α (HNF-1α) mRNA and protein levels and nuclear promoter complex expression were significantly reduced with curcumin treatment, suggesting that curcumin treatment regulates PCSK9 promoter activity through HNF-1α regulation (Table 2) [83].

Treatment of human L02 hepatocytes with curcumin (2.5 and 5 µM) resulted in increased genotoxic quinocetone (QCT)-induced cell viability and reduced QCT-induced DNA fragments and micronuclei formation (Table 2) [84]. Curcumin treatment reduced oxidative stress induced by QCT by reduced ROS production and increased SOD activity and GSH levels [84]. These data suggest that curcumin treatment prevents QCT-induced oxidative stress and cytotoxicity through antioxidative effects.

Treatment of primary mice hepatocytes with curcumin (25 µM) for 6 h resulted in significantly increased insulin signaling and reduced glucose production [85]. Insulin signaling improvements was indicated by increased Akt S437 phosphorylation with curcumin pre-treatment. Hepatic glucose production was inhibited with curcumin treatment and further repressed with the addition of insulin. In addition, curcumin treatment increased fibroblast growth factor 21 (FGF21) protein levels, a novel hepatic hormone hypothesized to be correlated with improved insulin sensitivity (Table 2) [85].

In another study by Dai et al. (2016), pre-treatment of L02 cells with curcumin (5 µM) for 2 h resulted in significantly reduced QCT-induced oxidative stress, mitochondrial dysfunction and apoptosis [86]. In addition, inducible nitric oxide synthase (iNOS) mRNA and activity and nitric oxide (NO) production were decreased with curcumin pre-treatment, indicating reduced oxidative stress. NF-kB mRNA level was reduced, while Nrf2 and heme oxygenase-1 (HO-1) mRNA levels were increased with curcumin treatment, suggesting reduced inflammatory and oxidative injury (Table 2) [86].

Treatment of HepG2 cells with KBH-1, a herbal mixture that is composed of one-third of curcumin (30 µM), for 24 h resulted in significantly improved lipid accumulation [87]. Treatment with KBH-1 reduced lipogenesis factors, sterol regulator element binding protein-1c (SREBP-1c), stearoyl-CoA desaturase-1 (SCD-1) and CD36 mRNA levels, while lipolysis factors, ACOX1, carnitine palmitoyltransferase I (CPT-1) and PPARα mRNA levels were increased, suggesting improved fatty acid catabolism [87]. The up-regulation of key lipolysis gene (CPT-1) indicate improved transport and oxidation of fatty acids. In addition KBH-1 treatment increased phosphorylated AMPK and ACC protein levels, suggesting that KBH-1 inhibited lipid accumulation by increased AMPK phosphorylation (Table 2) [87].

Treatment of primary mouse hepatocytes with curcumin (1 µM) for 4 h resulted in significantly increased lipogenesis [88]. A key lipogenic transcription factor, carbohydrate-response element-binding protein (ChREBP), is upregulated during acute elevations of plasma insulin levels to counteract postprandial rise in plasma glucose levels, resulting in the activation of downstream enzymatic targets to shift the glycolytic end-products towards lipogenesis [88]. Curcumin treatment increased ChREBPα mRNA level and upregulated the mRNA levels of genes that encode lipogenic enzymes (L-type pyruvate kinase (Lpk), Fas, Acc1, Scd1 and cytosolic malic enzyme 1 (Me1)). Curcumin treatment also stimulates ChREBP expression at the transcription level, as shown with increased ChREBPα-LUC fusion gene construct activity [88]. The increased ChREBPα-LUC activity by curcumin was blocked by MEK/ERK inhibition (PD98059), while Akt inhibition (AKTi) had no effect. In addition, curcumin treatment attenuated the binding of transcriptional repressor Oct-1 to the ChREBPα gene promoter. Additionally, Pak1-/- hepatocytes or Pak1 inhibited (IPA3) hepatocytes resulted in significantly reduced curcumin-stimulated ChREBPα mRNA and increased Oct-1 protein levels, suggesting that Pak1 is a necessary mediator in curcumin’s effect on ChREBPα expression (Table 2) [88].

Treatment of HepG2 and primary mouse hepatocytes with curcumin (2 µM) for 6 h resulted in significantly reduced fibroblast growth factor 21 (Fgf21) resistance, a hepatokine that is paradoxically found to be elevated with obesity and diabetes, while having attenuated action [89]. Curcumin treatment increased Fgf21 and upstream Fgf21 activator PPARα mRNA levels [89]. Additionally, curcumin treatment increased Fgf21-LUC reporter activity and improved PPARα binding to the Fgf21 gene promoter, indicating increased activity. These results were attenuated with co-treatment of PPARα antagonist GW6471, resulting in significantly reduced Fgf21 mRNA and protein levels, indicating that curcumin acts through PPARα to regulate Fgf21 activity (Table 2) [89].

Treatment of bisphenol A (BPA)-stimulated insulin resistant HepG2 cells with curcumin (5 µM) for 5 days resulted in significantly attenuated BPA-induced insulin resistance and oxidative stress [90]. Curcumin treatment increased BPA-stimulated glucose uptake and phosphorylated IR (Y1316) and Akt (S307/S473) protein levels, while phosphorylated IRS1 (S307) protein levels were reduced, indicating reduced insulin resistance. BPA-stimulated oxidative stress MDA and pro-inflammatory cytokine (IL-6 and TNF-α) levels were reduced with curcumin treatment. Additionally, important inflammatory and stress signaling molecules, JNK, p38, IKKβ and IκB-α phosphorylated, had reduced protein levels with curcumin treatment, suggesting that curcumin may act by regulating JNK/p38/NFκB signaling regulation (Table 2) [90].

Treatment of human LX-2 HSCs with curcumin (40 µM) for 24 h resulted in significantly increased lipocyte phenotype and reduced fibrotic myofibroblastic phenotype, a phenotype induced with diabetes and liver injury (Table 2) [91]. Curcumin treatment increased lipid droplet abundance and triglyceride content, a main component of lipid droplets. Curcumin treatment increased Nrf2 protein levels and nuclear translocation, suggesting increased activity. In addition, PPARα protein levels were reduced, while C/EBPα, PPARγ, retinoid X receptor (RXR)α and RXRβ protein levels were increased, suggesting favoured lipocyte phenotype. The presence of Nrf2 silencing RNAs abolished the effect of curcumin treatment on lipocyte proteins and lipid droplet abundance [91]. These data indicate that Nrf2 is required for curcumin’s regulation of lipid metabolism and induction of the lipocyte phenotype.

In a study by Yan et al. (2018), treatment of primary hepatocytes with curcumin (10 µM) for 24 h resulted in increased palmitate-induced cholesterol 7A1-hydroxylase (CYP7A) and cytochrome P4503A (CYP3A), both involved in bile acid regulation and are associated with improved liver function [92]. In addition, curcumin treatment reduced palmitate-induced SREBP-1c protein levels, indicating reduced steatosis and fatty acid synthesis (Table 2) [92].

Treatment of HepG2 and primary mouse hepatocytes with curcumin (10 µM) resulted in significantly reduced palmitate- and glucagon-stimulated glucose production (Table 2) [93]. Hepatic gluconeogenesis is initiated by cyclic adenosine monophosphate/protein kinase A (cAMP/PKA) signaling. The palmitate-induced cAMP levels were reduced, while phosphodiesterase 4B (PDE4B) activity was increased with curcumin treatment, suggesting reduced cAMP signaling and reduced gluconeogenesis [93]. In addition, phosphorylated AMPK protein level was increased with curcumin treatment, and the induction of PDE4B by curcumin was abolished in the presence of AMPK siRNA, indicating that AMPK is required for PDE4B regulation. Mitochondrial complex I activity, acetyl CoA accumulation and PDK4 mRNA levels were reduced with curcumin treatment, suggesting reduced mitochondrial oxidation. Additionally, curcumin treatment reduced pyruvate dehydrogenase (PDH) activity and inhibited pyruvate carboxylase (PC) protein level, indicating further indicating reduced gluconeogenesis [93].

Treatment of Buffalo rat liver cell line (BRL-3A) and HepG2 hepatocytes with curcumin (2.5 µM) for 8 and 12 h, respectively, resulted in significantly reduced fructose-induced inflammation [94]. Curcumin treatment also reduced ROS production and hydrogen peroxide (H_2_O_2_) levels, indicating reduced oxidative stress and inflammation. In addition, inflammatory (IL-1β and caspase 1) and oxidative stress (thioredoxin interacting protein (TXNIP)) protein levels were reduced with curcumin treatment [94]. Curcumin treatment significantly increased microRNA-200a (miR-200a) levels, while apoptosis-associated speck-like protein containing CARD (ASC) and NOD-like receptor pyrin domain containing (NLRP3) protein levels and NLRP3 inflammasome activity were inhibited, suggesting that miR-200a may be a biomarker of fructose-induced hepatic inflammation and that curcumin protects against inflammation by regulating TXNIP/NLRP3 inflammasome activity (Table 2) [94].

In a study by Lee et al. (2019), treatment of HepG2 cells with curcumin loaded nanoemulsion (TE-NE; 10 µM) for 24 h resulted in significantly reduced palmitate-induced lipotoxicity [95]. TE-NE treatment reduced the palmitate-induced lipid vacuole accumulation and ROS production, compared to curcumin treatment alone. In addition, SREBP-1, PPAR-γ2, cleaved caspase-3 and PARP levels were reduced with TE-NE treatment [95]. These data suggest that the use of nanoemulsions can improve curcumin’s efficacy and results in significantly greater reduced cellular damage induced by palmitate (Table 2).

Treatment of HepG2 cells with a19, a resveratrol-curcumin hybrid, for 1 h resulted in significantly reduced palmitate-induced inflammatory injury. Pro-inflammatory, TNF-α, IL-6, IL-1β and Cox-2 mRNA levels were reduced with a19 treatment, suggesting reduced inflammation [96]. In addition, palmitate-stimulated adhesion molecules ICAM, VCAM-1 and MCP-1 mRNA levels and fibrosis α-SMA, COL-1, COL-4 and TGF-β mRNA levels were reduced with a19 treatment, indicating reduced hepatic steatosis and fibrosis (Table 2) [96].

Overall, these studies suggest that treatment of hepatocytes with curcumin results in reduced cell proliferation and lipid deposition/lipogenic gene (Lpk, Fas, Acc1, Scd1 and Me1) expression. Curcumin treatment significantly reduced gluconeogenesis and increased glucokinase activity and glucose-6-phosphate levels. In addition, curcumin treatment significantly reduced inflammation cytokine (IL-6, IL-1β and TNF-α) and fibrosis gene (α-SMA, collagen and fibronectin) expression and increased antioxidant (SOD, catalase, glutathione and GSH) activities, resulting in decreased oxidative stress (Table 2).

### 2.3. Effects of Curcumin: In Vitro Muscle Cell Studies

Treatment of C2C12 mouse myoblasts with curcumin (40 µM) for 1 h resulted in increased glucose uptake and GLUT4 translocation to the cell surface [97]. In addition, curcumin treatment significantly increased the phosphorylation of AMPKα and ACC, while the phosphorylation of Akt was unchanged. Synergistically, the co-treatment of curcumin and insulin increased phospho-AMPKα, phospho-ACC and phospho-Akt protein levels, and increased the membrane expression of GLUT4. Although, the effects of curcumin could be independent to insulin, curcumin’s effects can be enhanced by insulin to further its effects (Table 3) [97].

In a study by Deng et al. (2012), treatment of palmitate-induced insulin resistant C2C12 muscle cells with curcumin (20 µM) for 2 h resulted in increased glucose uptake [98]. In addition, curcumin treatment improved insulin signaling with reduced palmitate-stimulated phosphorylated IRS-1 (S307) and ACC protein levels, while phosphorylated Akt, ERK1/2 and p38 protein levels were increased (Table 3) [98].

Treatment of C2 murine myoblasts with increasing dosages of curcumin (1–30 µM) for 24 h resulted in increased cell viability, however when treated at 50 µM, the C2 viability levels dropped by 54%, suggesting toxic effects [99]. In addition, curcumin treatment (50 µM) significantly increased phosphorylated JNK and poly(ADP-ribose) polymerase (PARP) fragment protein levels, suggesting increased apoptosis (Table 3) [99]. These data suggest that high dose of curcumin to C2 myoblasts produce pro-oxidant and apoptotic effects and highlights the importance of dose and toxicity.

Combined treatment of C2C12 myotubes with curcumin (10 µM) and eicosapentaenoic acid (EPA; 50 µM) for 24 h resulted in significantly reduced proteolysis-inducing factor (PIF)- and TNF-α-induced protein degradation (Table 3) [100]. The activity of chymotrypsin-like enzyme, as an indicator of proteasome activity, was significantly increased by TNF-α and PIF, and was attenuated with curcumin and EPA co-treatment. In addition, curcumin and EPA significantly increased protein synthesis. PIF and TNF-α reduced myotube diameter (13 µm) and was attenuated with EPA and curcumin co-treatment (17 µm) to levels similar to control myotubes [100].

In a study by Sadeghi et al. (2018), pre-treatment of C2C12 muscle cells with curcumin (40 µM) for 1 h resulted in significantly reduced palmitate-induced inflammation, with decreased TNF-α and IL-6 mRNA and cytokine levels [101]. Curcumin treatment reduced ROS production and the level of superoxide. In addition, the phosphorylation of JNK and IKKα-IKKβ protein levels were reduced with curcumin treatment (Table 3) [101].

Curcumin is not water soluble, a factor that contributes to its low bioavailability. In a study by Chauhan et al. (2018), treatment of palmitate-stimulated insulin resistant L6myc myotubes with curcumin loaded chitosan nanoparticles (CCN; 25 µM) for 16 h resulted in increased GLUT4 translocation to the plasma membrane [102]. Insulin-stimulated GLUT4 translocation and phospho-Akt and GSK-3β protein levels were further increased with CCN treatment [102]. Pro-inflammatory palmitate-induced TNF-α, IL-6 and MCP-1 cytokine levels were reduced, while anti-inflammatory IL-10 cytokine level was increased with CCN treatment, suggesting anti-inflammatory effects of CCN (Table 3) [102].

Overall, these studies suggest that treatment of skeletal muscle cells with curcumin resulted in improved glucose uptake and GLUT4 translocation. Curcumin treatment exerts anti-inflammatory effects by reducing pro-inflammatory (TNF-α, IL-6 and MCP-1) mRNA and cytokine levels and increasing anti-inflammatory IL-10 cytokine levels. In addition, curcumin treatment protects skeletal muscle cells from protein degradation by anti-catabolic effects that improve protein synthesis (Table 3).

### 2.4. Effects of Curcumin: In Vitro Pancreatic Cell Studies

Treatment of Sprague–Dawley isolated pancreatic islets with curcumin (10 µM) for 30 min resulted in increased insulin release in the presence of 4 mM glucose [103]. The open channel probability of volume-regulated anion channels in islet cells were activated with curcumin treatment. Curcumin treatment caused a rapid, progressive and irreversible increase in membrane conductance, suggesting a non-specific disruption of plasma membrane integrity. which was determined to be due to increased channel open probability. Furthermore, curcumin treatment increased glucose-induced depolarization of the cell membrane potential. These effects on depolarization were attenuated with the absence of intracellular chloride ions, suggesting that islet cell depolarization and electrical activity in response to curcumin is dependent on intracellular [Cl-] (Table 4) [103]. These data suggest that curcumin stimulates insulin release through the regulation of depolarization and electrical activity dependent on chloride ions.

In a study by Pugazhenthi et al. (2007), treatment of MIN6 cells with curcumin (20 µM), DMC (20 µM), or BDMC (20 µM) for 24 h resulted in increased β-cell stress defense and survival [104]. Diabetes can lead to oxidative stress-induced β-cell loss, resulting in reduced insulin secretion and increased glucose toxicity. Curcumin, DMC and BDMC treatment increased antioxidant HO-1 mRNA levels and promoter expression, suggesting improved stress protection [104]. This increased in HO-1 promoter expression was dependent on the presence of antioxidant response element (ARE) sites containing enhancer regions (E1 and E2) and Nrf2 expression. The mRNA levels of HO-1, γ-glutamylcysteine ligase modulatory subunit (GCLM) and NAD(P)H:quinone oxidoreductase (NQO1) were increased with curcuminoids treatment [104]. Curcumin, DMC and BDMC increased HO-1 promoter and protein level was inhibited by wortmannin (PI3K inhibitor; 40%), Akt inhibitor IV (Akt inhibitor; 55%) and rottlerin (PKCδ inhibitor; 25%), indicating that curcuminoid HO-1 expression is regulated by PI3K/Akt (Table 4) [104].

Pretreatment of primary mouse pancreatic islets with curcumin (10 µM) for 24 h resulted in increased streptozotocin (STZ)-induced islet viability and insulin secretion [105]. Curcumin treatment significantly reduced oxidative stress with decreased ROS production and MDA levels and increased Cu/Zn SOD levels. In addition, peroxynitrite, NO and activated PARP levels were reduced with curcumin treatment, suggesting increased cytoprotection (Table 4) [105].

An alternative treatment to T1DM than the conventional exogenous insulin injection is pancreatic islet transplantation. However, this procedure is limited by islet cryopreservation and recovery during the thawing process [117]. In a study by Kanitkar et al. (2008) presence of curcumin (10 µM) with primary murine islets during cryopreservation resulted in increased islet recovery and morphology [106]. Glucose responsiveness and insulin secretion were increased, and ROS produced was reduced in islets cryopreserved with curcumin. Heat shock protein 70 (Hsp70) and HO-1 levels were also increased in islets cryopreserved with curcumin (Table 4) [106].

Treatment of primary pancreatic islets with curcumin (10 µM) protected islets from cytokine (TNFα, IL-1β and IFNγ)-induced islet death and dysfunction by reduced ROS production [107]. Insulin secretion and mRNA levels were significantly increased with curcumin treatment. In addition, curcumin treatment reduced cytokine-induced NF-κB translocation and inhibited the phosphorylation of IκBα (Table 4) [107].

Isolated human islets treated with curcumin (40 µM), DMC (40 µM) and BDMC (40 µM) for 24 h resulted in increased insulin secretion, however glucose-stimulated insulin secretion was not increased [108]. In addition, HO-1, GCLM and NQO1 mRNA and protein levels were increased with curcumin and curcuminoids treatment, suggesting increased survival and function (Table 4) [108].

In a study by Abdel Aziz et al. (2010), treatment of isolated rat islets with curcumin (10 µM) for 4 h resulted in significantly increased in insulin secretion and HO-1 mRNA and activity [109]. The curcumin increase in insulin secretion was reduced with stannous mesoporphyrin, HO inhibitor, suggesting that curcumin’s effect is mediated by HO-1 expression (Table 4) [109].

Treatment of β-Min6 and HP62 β-cells with curcumin (100 pM) significantly increased high glucose-induced insulin secretion and intracellular cAMP levels (Table 4) [110]. Three phosphodiesterase (PDE) isoforms, *Pde3b*, *Pde8a* and *Pde10a,* were significantly reduced with curcumin treatment, indicating enhanced β-cell function. In addition, curcumin treatment reduced low and high glucose-induced PDE activity, as determined by decreased cAMP degradation to 5′-AMP [110].

Treatment of INS-1 cells with curcumin for 24 h resulted in increased glucose-induced insulin secretion [111]. Curcumin treatment increased GLUT2 and insulin mRNA, tyrosine phosphorylation of InsR and IRS-1. In addition, curcumin treatment significantly increased high glucose treated phosphorylated PI3Kp85 and Akt protein levels and increased the association of PI3K to IRS1. Curcumin treatment significantly increased glucokinase (GCK) and pancreatic and duodenal homeobox-1 (PDX-1) protein levels (Table 4) [111].

Treatment of isolated male Wistar rat pancreatic islets with curcumin (20 µM) for 24 h resulted in increased STZ-induced cell viability and GLUT2 expression [112]. Curcumin treatment attenuated the STZ-reduced phosphorylated PI3Kp85, Akt and GSK3β protein levels, indicating improved insulin signaling. In addition, pro-inflammatory transcription factor NF-κB nuclear protein level and NO production was significantly reduced, while antioxidant transcription factor (Nrf-2), responsible for the activation of antioxidant response, and HO-1 protein levels were increased with curcumin treatment [112]. Pro-apoptotic Bax, cleaved caspase-12, -3, -8 and -9 protein levels were significantly reduced with curcumin treatment, while anti-apoptotic Bcl-2 protein levels were increased, suggesting curcumin treatment protects against STZ-induced apoptosis (Table 4) [112].

MIN6 pancreatic β-cells treated with curcumin (10 µM) for 24 h resulted in improved cell viability and increased glucose-induced insulin secretion [113]. Palmitate-induced apoptosis, caspase-3 and caspase-9 activities, Bcl/Bax ratio and ROS production was significantly reduced with curcumin treatment. Lipid peroxidation index MDA protein levels were also reduced with curcumin treatment [113]. In addition, antioxidant enzyme (SOD, catalase, GPx and glutathione) activities were increased with curcumin treatment. These effects were suggested to be mediated by Akt/FoxO1 activation due to increased phosphorylated Akt (S473) and FoxO1 (S256) protein levels with curcumin treatment (Table 4) [113].

Treatment of INS-1 insulinoma cells with curcumin conjugated to R3V6 peptide micelles (10:3 weight ratio; 22 mg/L) for 24 h resulted in increased curcumin cellular uptake compared to curcumin treatment alone [114]. Hypoxic-induced cell viability was more significantly increased by both R3V6-curcumin than curcumin treatment alone. In addition, R3V6-curcumin treatment reduced hypoxic-induced apoptosis and ROS production (Table 4) [114].

Treatment of isolated pancreatic islets with tetrahydrocurcumin (THC; 12.5 µM), a metabolite of curcumin, for 24 h resulted in significantly increased glucose-induced insulin secretion [115]. Curcumin treatment also significantly increased cytokine (TNF-α, interferon-β, and IL-1β) treated glucose-induced insulin resistance, glutathione levels and reduced NO production. In addition, apoptosis, active caspase-9, activated caspase-3 and Bax protein levels were reduced with curcumin treatment, while anti-apoptotic Bcl-2 protein levels were increased, suggesting protection against cytokine-induced apoptosis (Table 4) [115].

In a study by Kose et al. (2019), pre-treatment of MIN6 cells with curcumin (20 µM) for 24 h resulted in reduced ferroptosis, programmed cell death induced by lipid peroxidation [116]. Curcumin treatment increased erastin-induced cell viability dose- and time-dependently. In addition, iron levels, glutathione peroxidase 4 (GPX4) and lipid peroxidation were reduced, while glutathione levels were increased with curcumin treatment (Table 4) [116].

Overall, these studies indicated that treatment of pancreatic islets with curcumin and curcuminoids resulted in increased insulin secretion and islet recovery. HO-1 mRNA and protein levels and promoter activity were significantly increased with curcumin treatment indicating reduced apoptosis and oxidative stress, in conjunction with increased antioxidant enzyme activities. These effects are suggested to be due to insulin signaling and PDE/cAMP regulation (Table 4).

## 3. Conclusions and Future Directions

**Figure 2:**
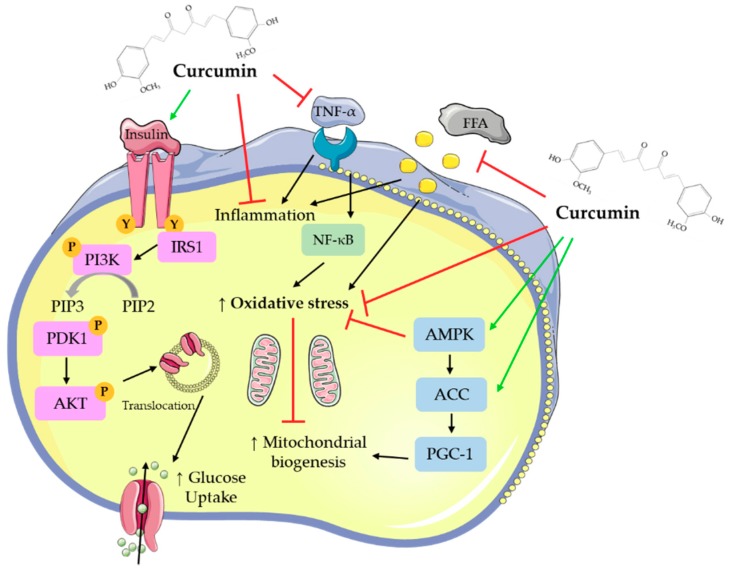
Cellular effects of curcumin on muscle and fat cellular signaling molecules. The figure was created based on the data of the studies [40,41,43,44,45,46,57,67,73,97,98,101,102]. AKT: protein kinase B; PIP3: phosphatidylinositol-3,4,5-triphosphate; PIP2: phosphatidylinositol 4,5-bisphosphate; ERK: extracellular signal-regulated kinase; PI3K: phosphoinositide 3-kinase; IRS1: insulin receptor substrate 1; TNF-α: tumor necrosis factor- α; AMPK: AMP-activated protein kinase; NF-κB: nuclear factor kappa-light-chain-enhancer of activated B cells; ACC: acetyl-CoA carboxylase; PGC-1: peroxisome proliferator-activated receptor gamma co-activator 1; FFAs: free fatty acids.

Overall, all available in vitro studies examining the effects of curcumin indicate increased glucose uptake and utilization by skeletal muscle cells and adipocytes, reduced hepatocyte lipid deposition, and inhibition of gluconeogenesis. Pancreatic beta cell function was improved by curcumin treatment. Figure 2 was created based on the in vitro studies presented in the current review and summarizes the main effects of curcumin.

Treatment of adipocytes with curcumin (5–100 μM) for up to 72 h resulted in reduced adipocyte differentiation, and lipid accumulation. Macrophage infiltration of adipocytes was reduced with curcumin treatment, as well as pro-inflammatory cytokine production and signaling. In addition, curcumin treatment suppressed adipogenic gene expression, while mitochondrial biogenesis and membrane potential were improved.

Treatment of hepatocytes with curcumin (5–100 μM) for up to 5 days resulted in reduced lipid deposition/lipogenic gene expression. Curcumin treatment significantly reduced inflammatory cytokine and fibrosis gene expression and increased antioxidant activities, resulting in decreased oxidative stress. In addition, gluconeogenesis was reduced, while glucokinase activity and glucose-6-phosphate levels were increased with curcumin treatment.

Skeletal muscle cells treated with curcumin (10–50 μM) for up to 24 h had improved glucose uptake and GLUT4 translocation. Curcumin treatment exerted anti-inflammatory effects, it reduced pro-inflammatory mRNA and cytokine levels and increased anti-inflammatory cytokine levels.

Treatment of pancreatic islets with curcumin and curcuminoids (100 pM–57 μM) for up to 24 h resulted in increased insulin secretion and islet cell recovery. HO-1 promoter activity and mRNA and protein levels and antioxidant enzyme activities were significantly increased with curcumin treatment indicating reduced apoptosis and oxidative stress.

The in vitro studies presented in the current review may have used different curcumin concentrations and different treatment times. A careful examination of the studies revealed that overall, the common curcumin concentrations used were in the micromolar level, with most of the studies using 10–20 μM curcumin.

Discrepancies are shown regarding curcumin’s effects on adipocytes. As mentioned in Section 2.1, studies by Green et al. (2014) [56] and Zhang et al. (2016) [60] showed that treatment of adipocytes with curcumin resulted in reduced insulin-stimulated glucose uptake and GLUT4 translocation to the plasma membrane [56,60]. These results are in contrast to other studies performed on adipocytes, demonstrating antidiabetic effects with curcumin treatment. Therefore, more studies are needed to examine in more detail the effects of curcumin on adipocytes.

Curcumin has the potential to attenuate inflammatory and oxidative stress diseases through increased antioxidant activities. A systematic and meta-analysis review by Wal et al. (2019) [118], found that curcumin blocked the oxidation process in mitochondria and reduced ROS and cytokine production and increased the activities of antioxidant enzymes [118]. Although increased antioxidant intake, such as curcumin, has been traditionally thought to result in increased health [118,119,120], this concept has been recently challenged [121,122]. In the review by Halliwell (2013) [121], administration of large doses of dietary antioxidants to humans with oxidative diseases had little to no preventative or therapeutic effects. Instead, administration of weak pro-oxidants may have a greater effect on oxidative disease treatment and prevention [121]. In 2012, the United States Department of Agriculture (USDA) decided to remove the use of the oxygen radical absorbance capacity (ORAC) table, which indicates the antioxidant power of bioactive compounds, including polyphenols such as curcumin. The decision was due to the belief that in vitro measurements of antioxidant capacity have no relevance to the effects of specific bioactive compounds on human health and that the ORAC values were routinely misused by manufacturing companies to promote their products. Clearly, the mechanisms of action of antioxidants, including curcumin, and the methodology used to quantify the effects require extensive research before human supplementation is recommended.

As mentioned in the introduction, a search of the literature resulted in many studies focusing on the antidiabetic properties of curcumin and we have prepared an additional review manuscript focusing on the in vivo animal studies and clinical trials titled, Antidiabetic properties of curcumin II: Evidence from in vivo studies. Although all the available in vitro and in vivo studies suggest a strong potential of curcumin to be used in the treatment against insulin resistance and T2DM, we acknowledge the need for further clinical studies. Investigations focusing on the effective dose of curcumin in humans as well as the detailed effects on plasma glucose, lipid, insulin and HbA1c levels should be further explored.

Overall, the cellular effects of curcumin are widespread, and the low toxicity of the molecule makes it a prime candidate for medicinal use against insulin resistance and T2DM.

## Figures and Tables

**Table 1:** Effects of curcumin: in vitro adipocyte studies.

Cell	Curcumin Concentration/Duration	Effect	Reference
Human adipocytes	20 µM; 14 days	↑ Adipocyte differentiation ↑ Glucose elevation levels ↑ PPAR-γ activity	[40]
Human adipocytes	20 µM; 14 days	↑ Adipocyte differentiation ↑ Glucose elevation levels ↑ PPAR-γ activity	[41]
Raw 264.7 macrophages and 3T3-L1 adipocytes	10 µM; 24 h	↓ Macrophage migration ↓ MCP-1 ↓ TNF-α ↓ NO	[42]
3T3-L1 adipocytes	20 µM; 20 min and 62 h	↓ TNFα-activated NF-κB signaling ↓ IκB degradation ↓ NF-κB nuclear transition ↓ TNFα, IL-1β and IL-6 mRNA ↓ COX-2 mRNA ↓ IL-6 and PGE_2_ secretion	[43]
3T3-L1 adipocytes	5–20 µM; 24 h	↓ Adipogenesis ↓ Adipocyte differentiation ↓ Apoptosis ↑ Phospho-AMPK) ↑ Phospho-ACC ↓ GPAT-1 mRNA ↑ CPT-1 mRNA	[44]
3T3-L1 adipocytes	5–20 µM; 24 h	↑ Glucose uptake ↓ TNF-α mRNA ↓ IL-6 mRNA ↓ NF-κBp65 protein ↓ Phospho-JNK ↓ Phospho-ERK ↓ Phospho-p38	[45]
3T3-L1 adipocytes	10–50 µM; 8 days	↓ Adipocyte differentiation ↑ Phospho-AMPK ↓ PPARγ activity	[46]
3T3-L1 adipocytes	10–25 µM; 48 h	↓ Adipocyte differentiation ↓ Phospho-JNK ↓ Phospho-ERK ↓ Phospho-p38 ↑ β-catenin nuclear translocation ↓ CK1α, GSK-3β and Axin mRNA ↓ AP2 mRNA ↓ Wnt10b, Fz2 and LRP5 mRNA ↑ c-Myc and cyclin D1 mRNA	[47]
Rabbit subcutaneous adipocytes	5–20 µM; 24 h	↑ Cholesterol efflux ↑ PPARγ, LXRα and ABCA1 mRNA	[48]
3T3-L1 adipocytes and human primary preadipocytes	5–30 µM; 6 days	↓ Adipocyte differentiation ↓ Lipid accumulation ↓ C/EBPβ, C/EBPα, PPARγ, leptin, adiponectin and resistin mRNA ↓ MCE ↓ S to G2/M phase transition ↓ Cyclin A and CDK2 protein	[49]
3T3-L1 adipocytes	20 µM; 48 h	↓ Adipocyte differentiation ↓ Lipid accumulation ↓ Fatty acid synthase ↓ Acetyl-CoA and malonyl-CoA ↓ PPARγ and CD36 mRNA	[50]
3T3-L1 adipocytes and preadipocytes	20 µM; 48 h	↓ Adipocyte differentiation ↓ C/EBPβ, C/EBPα, PPARγ, leptin, adiponectin and resistin mRNA	[51]
3T3-L1 adipocytes	10–20 µM; 24 h	↓ Lipolysis ↓ Phospho-ERK1/2 ↓ Phospho-perilipin ↑ Perilipin ↓ Hormone-sensitive lipase translocation	[52]
3T3-L1 adipocytes	10 and 50 µM; 24 h	↓ LPS-induced leptin levels ↓ LPS-induced IL-6 secretion	[53]
Bone marrow mesenchymal stem cells (MSCs)	15 µM; 10 days	↓ Adipocyte differentiation ↓ PPARγ2 mRNA ↓ C/EBPα mRNA	[54]
Human adipocytes	15 µM; 24 h	↓ Oxidative stress ↓ ROS production	[55]
Primary rat adipocytes	20 µM; 45 min	↓ Insulin-stimulated glucose uptake	[56]
3T3-L1 adipocytes	20 µM; 24 h	↓ Cell injury ↓ ROS production ↓ HIF1α mRNA ↓ Lactate release ↓ Glycerol release ↓ Lipid peroxidation ↓ Protein oxidation ↑ SOD and CAT activities ↓ TNFα, IL-6, IL-1β and IFN-γ ↑ Mito biogenesis ↑ Mito membrane potential ↓ Mito superoxide production ↓ Transition pore permeability	[57]
3T3-L1 adipocytes	10 µM; 48 h	↓ Lipid accumulation ↓ PPARγ, aP2 and C/EBPα mRNA ↑ CPT-1, MCAD and VLCAD mRNA ↑ ACOX and thiolase mRNA ↑ PPARα reporter mRNA	[58]
3T3-L1 adipocytes	10–100 µM; 24–72 h	↓ Cell viability ↑ Apoptosis ↑ Bax ↓ Bcl-2 ↑ Cytochrome c release ↑ PARP cleavage	[59]
3T3-L1 adipocytes	0–50 µM; 0–24 h	↓ Glucose uptake ↓ Akt protein ↓ Glucose transporter (GLUT4) plasma membrane expression ↓ Trypsin-like peptidase activities ↓ p62 protein ↑ LC3-II protein ↑ LC3-II/LC3-I ratio	[60]
3T3-L1 adipocytes	10 µg/mL; 24 h	↑ Lipolysis ↓ Lipid accumulation ↑ Free fatty acid levels ↑ Glycerol levels ↑ Adipose triglyceride lipase mRNA ↑ Hormone-sensitive lipase mRNA ↓ Leptin levels	[61]
Primary adipocytes	20 µM; 8 days	↓ Adipogenesis ↓ Lipogenesis ↓ Protein levels associated with lipolysis ↓ Protein levels associated with fatty acid β-oxidation ↓ Protein levels associated with energy expenditure ↓ ALDH2, ATP5B and PRDX5 ↑ ACCA2, ACO2, ATP5H, CS, DLD, GLUD1, HADHA, HSPD1, Hsp10, HSPE1 and VDAC1 ↓ ANXA2 and HSP90 protein ↑ ATP5B, HSL, CA3 and MDH protein ↑ Ucp1, Ucp2 and Ucp3 mRNA	[62]
3T3-L1 adipocytes	20 µM; 72 h	↓ Adipocyte differentiation ↓ Ubiquitin proteasome activity ↓ Skp2 protein accumulation ↑ p27 protein accumulation ↑ G1 arrest ↓ p27 mRNA	[63]
3T3-L1 adipocytes	25 µM; 30 min	↓ Lipid accumulation ↓ MCE ↓ C/EBPα and PPARγ protein ↑ Cell cycle arrest ↓ Cyclin A, cyclin B and p21 protein ↓ Phospho-ERK ↓ Phospho-JNK ↓ Phospho-p38	[64]
3T3-L1 and primary adipocytes	20 µM; 6 days	↑ Brown fat phenotype ↑ Mitochondrial biogenesis ↑ Mitochondrial density ↑ PGC-1α, PPARγ, PRDM16 and Ucp1 protein ↑ PGC-1α mRNA ↑ Mito CPT1 protein ↑ Cytochrome c protein ↑ Phospho-AMPK ↑ AMPK	[65]
3T3-L1 adipocytes	20 µM; 24 h	↑ Glucose metabolism ↑ Lipid metabolism ↓ Glycerol release ↑ Glucose uptake ↑ PPARγ and C/EBPα mRNA ↑ PPARγ and C/EBPα protein	[66]
3T3-L1 adipocytes	20 µM; 24 h	↓ Glucose uptake ↑ Insulin sensitivity ↓ Leptin levels ↑ Adiponectin levels ↓ Resistin mRNA ↓ MCP-1 levels ↓ TLR-4 mRNA ↓ NF-kB p65 nuclear fraction ↓ Phospho-JNK1/2 ↓ Phospho-IRS-1(S) ↑ IRS-2 protein ↓ GLUT1 mRNA and protein ↑ GLUT4 mRNA and protein ↓ MMP-2, MMP-9 and VEGF protein ↓ Angpt4 protein level	[67]
3T3-L1 adipocytes	20 µM; 7 days	↓ Adipocyte differentiation ↓ Lipid content accumulation ↓ C/EBPα, αP2, SREBP1c and PPARγ mRNA ↓ FAS protein ↑ UCP1 and PGC-1α protein ↑ Oxygen consumption rate ↑ Cell cycle arrest ↓ Cyclin D1, cyclin D3, CDK2, CDK4 and CDK6 protein	[68]
Primary and mouse brown adipocyte cell (mBAC) adipocytes	2 µM; 10 h	↓ WAT inflammation ↑ UCP1, PPARα and PGC-1α mRNA (*primary*) ↑ UCP1, PPARα, PPARγ and PGC-1α mRNA (*mBAC*)	[69]
Human bone marrow MSCs	10 µM; 10 days	↓ Adipocyte differentiation ↓ Lipid content accumulation ↓ PPARγ, C/EBPα, FABP4 and KLF15 mRNA and protein	[70]

**Table 2:** Effects of curcumin: in vitro hepatocyte studies.

Cell	Curcumin Concentration/Duration	Effect	Reference
Hepatic stellate cells	30 µM; 2 h	↓ Cell proliferation ↑ Apoptosis ↓ α1(I) collagen, α-SMA and fibronectin mRNA ↑ p21 and p27 protein ↑ Caspase-3 activity ↓ Bcl-2 mRNA ↑ NF-κB activity	[71]
Primary mice isolated hepatocytes	25 µM; 120 min	↓ Hepatic glycogenolysis ↓ Hepatic gluconeogenesis ↓ G6Pase activity ↓ PEPCK activity ↑ Phospho-AMPK	[72]
H4IIE rat hepatoma and Hep3B human hepatoma cells	2–50 µM; 30 min	↓ Hepatic gluconeogenesis ↓ PEPCK activity ↓ G6Pase activity ↑ AMPK activation	[73]
Hepatic stellate cells	0–30 µM; 1 h	↓ HSC activation ↑ HSC apoptosis ↓ PDGF-βR and EGFR ↓ α-SMA and α-II-collagen ↑ PPARγ ↓ Bcl-2 ↑ Bax ↓ ROS levels ↑ GSH protein ↑ GSH/GSSG ratio ↑ GCL activity ↓ Phospho-InsR, -ERK1/2, -JNK, -PI3K and -Akt	[74]
Isolated rat hepatocytes	1 and 10 µM; 30 min	1 µM ↓ Lipid peroxidation ↓ Cytochrome c translocation 10 µM ↑ Oxidative stress ↑ Hepatocytoxicity ↑ Necrosis ↑ Apoptosis ↑ Glutathione depletion ↑ Caspase-3 activity	[75]
Rat HSCs and immortalized human hepatocytes	20 µM; 1 h	↓ Glucose levels ↓ GLUT4 membrane translocation ↑ Glucokinase activity ↑ Glucose-6-phosphate levels ↓ Phospho-IRS-1 ↓ Phospho-PI3K ↓ Phospho-Akt	[76]
Huh7 cells	20 µM; 48 h	↑ PON1 transactivation ↑ PON1 mRNA and protein	[77]
L02 hepatocytes	-	↓ Oxidative stress ↓ Insulin resistance ↓ ROS production ↓ Glutathione depletion ↓ MDA levels ↓ LDH activity ↓ AST activity	[78]
Primary rat hepatocytes	10 µM; 12 h	↓ Lipo-apoptosis ↓ ROS production ↓ ATP depletion ↓ PEPCK levels ↓ G6Pase levels ↑ Mitochondrial DNA ↑ PGC1α, NRF1 and Tfam mRNA ↑ MMP ↓ NF-kB p65 mRNA	[79]
AML-12 cells	50 µM; 2 h	↑ Insulin response ↑ Phospho-PKB ↓ ROS levels ↓ MDA levels ↓ Phospho-JNK ↓ Phospho-p38	[80]
Primary rat hepatocytes	15 µM; 24 h	↓ Lipid formation ↑ APOBEC-1 mRNA and protein ↑ ApoB mRNA editing ↓ Apo-B lipid formation	[81]
HepG2 cells	30 µM; 2 h	↓ Cholesterol cell content ↓ Endosome/lysosome size and localization ↑ Cholesterol β-hexosaminidase enzyme level ↑ Lysosomal β-hexosaminidase enzyme level ↑ Flotillin-2-positive granules ↑ CD63-positive granules	[82]
HepG2 cells	5–20 µM; 24 h	↑ Hypolipidemic activity ↑ LDL uptake ↑ LDL receptor expression ↑ LDL receptor activity ↓ PCSK9 mRNA and protein ↓ PCSK9 promoter activity ↓ HNF-1α mRNA and protein ↓ HNF-1α nuclear promoter	[83]
L02 cells	2.5 and 5 µM; 2 h	↓ Oxidative stress ↑ Cell viability ↓ DNA-fragments ↓ Micronuclei formation ↓ ROS production ↑ SOD activity ↑ GSH levels	[84]
Primary mice hepatocytes	25 µM; 6 h	↓ Glucose production ↑ FGF21 protein ↑ Phospho-Akt	[85]
L02 cells	5 µM; 2 h	↓ QCT-induced inflammatory response ↓ QCT-induced oxidative stress ↓ Mitochondrial dysfunction ↓ Apoptosis ↓ iNOS activity ↓ NO production ↓ NF-κB mRNA ↑ Nrf2 and HO-1 mRNA	[86]
HepG2 cells	30 µM; 24 h	↓ Hepatic steatosis ↑ Leptin resistance ↓ Lipid deposition ↓ SREBP-1c, SCD-1 and CD36 mRNA ↑ ACC, ACOX1, CPT-1 and PPARα mRNA ↑ Phospho-AMPK ↑ Phospho-ACC	[87]
Primary mouse hepatocytes	1 µM; 4 h	↑ Lipogenesis ↑ ChREBP activation ↑ ChREBP mRNA ↑ Lpk, Fas, Acc1, Scd1 and Me1 mRNA ↑ ChREBP-LUC activity ↓ Oct-1-ChREBP binding	[88]
HepG2 and primary mouse hepatocytes	2 µM; 6 h	↓ Fgf21 resistance ↑ Fgf21 activity ↑ Fgf21 mRNA ↑ PPARα mRNA ↑ Fgf21-LUC activity ↑ PPARα-Fgf21 binding	[89]
HepG2 cells	5 µM; 5 days	↓ Insulin resistance ↑ Glucose consumption ↓ Oxidative stress ↑ Phospho-IR ↑ Phospho-Akt ↓ MDA levels ↓ IL-6 levels ↓ TNF-α levels ↓ Phospho-JNK and p38 ↓ Phospho-IKKβ and IκB-α	[90]
Human LX-2 HSCs	40 µM; 24 h	↓ Fibrotic myofibroblastic phenotype ↑ Lipocyte phenotype ↑ Lipid droplet abundance ↑ Lipid metabolism ↑ Triglyceride content ↑ Nrf2 protein and nuclear translocation ↑ C/EBPα, PPARγ, RXRα and RXRβ protein ↓ PPARα protein	[91]
Primary mice hepatocytes	10 µM; 24 h	↓ Palmitate-induced steatosis ↑ CYP7A and CYP3A protein ↓ SREBP-1c protein	[92]
HepG2 cells and primary mice hepatocytes	10 µM; 30 and 120 min	↓ Glucose production ↓ Mitochondrial complex I activity ↓ cAMP accumulation ↑ PDE4B induction ↑ Phospho-AMPK ↓ Acetyl CoA accumulation ↓ PDK4 mRNA ↓ PDH activity ↓ PC protein	[93]
BRL-3A and HepG2 cells	2.5 µM; 8 and 12 h	↓ Fructose-induced inflammation ↓ NLRP3 inflammasome activity ↑ miR-200a level ↓ NLRP3 and ASC protein ↓ Caspase-1 and IL-1β protein ↓ TXNIP protein ↓ ROS production ↓ H_2_O_2_ levels	[94]
HepG2 cells	10 µM; 24 h	↓ Lipotoxicity ↓ Lipid vacuole accumulation ↓ ROS production ↓ SREBP-1, PPAR-γ2 and PARP levels ↓ Cleaved caspase 3 levels	[95]
HepG2 cells	10 µM; 1 h	↓ Palmitate-induced inflammatory injury ↓ Palmitate-induced fibrosis ↓ TNF-α, IL-6, IL-1β and COX-2 mRNA ↓ ICAM, VCAM-1 and MCP-1 mRNA↓ α-SMA, COL-1, COL-4 and TGF-β mRNA ↓ α-SMA protein	[96]

**Table 3:** Effects of curcumin: in vitro muscle cell studies.

Cell	Curcumin Concentration/Duration	Effect	Reference
C2C12 cells	40 µM; 1 h	↑ Glucose uptake ↑ GLUT4 translocation ↑ Phospho-AMPK ↑ Phospho-ACC ↑ Insulin-induced phospho-Akt	[97]
C2C12 cells	20 µM; 2 h	↑ Glucose uptake ↓ Phospho-IRS-1 ↓ Phospho-ACC ↑ Phospho-Akt ↑ Phospho-ERK1/2 ↑ Phospho-p38	[98]
C2 murine myoblasts	50 µM; 24 h	↑ Apoptosis ↓ Cell viability ↑ PARP fragmentation ↑ Phospho-JNK	[99]
C2C12 cells	10 µM; 24 h	↓ Protein degradation ↓ Chymotrypsin-like enzyme activity ↑ Protein synthesis ↑ Myotube diameter	[100]
C2C12 cells	40 µM; 1 h	↓ Inflammation ↓ IL-6 mRNA and levels ↓ TNF-α mRNA and levels ↓ Phospho-IKKα-IKKβ ↓ Phospho-JNK ↓ ROS production	[101]
L6myc skeletal muscle cells	25 µM; 16 h	↑ GLUT4 translocation ↑ Phospho-Akt ↑ Phospho-GSK-3β ↓ TNF-α, IL-6 and MCP-1 levels ↑ IL-10 levels	[102]

**Table 4:** Effects of curcumin: in vitro pancreatic cell studies.

Cell	Curcumin Concentration/Duration	Effect	Reference
Sprague–Dawley rat pancreatic islets	10 µM; 30 min	↑ Insulin release ↑ Volume-regulated anion channel opening ↑ Depolarization ↑ Electrical activity	[103]
MIN6 cells and BALB/c mouse pancreatic islets	20 µM; 24 h	↓ Pancreatic cell death ↑ HO-1 mRNA and promoter ↑ GCLM mRNA ↑ NQO1 mRNA ↑ Nrf2 nuclear translocation	[104]
C57/BL6J mice pancreatic islets	10 µM; 24 h	↑ Islet viability ↑ Insulin secretion ↓ ROS production ↓ MDA levels ↑ SOD levels ↓ Peroxynitrite levels ↓ NO levels ↓ Activated PARP	[105]
Swiss albino mice pancreatic cells	10 µM; 24 h	↑ Islet recovery ↑ Glucose responsiveness ↑ Insulin secretion ↑ Morphology ↓ ROS production ↑ Hsp70 level ↑ HO-1 level	[106]
C57/BL6J mice pancreatic islets	10 µM; 24 h	↓ Islet death and dysfunction ↑ Insulin mRNA and level ↓ ROS production ↓ NF-κB translocation ↓ Phospho-IκBα	[107]
Human isolated islets	40 µM; 24 h	↑ Insulin secretion ↑ HO-1 ↑ NQO1 ↑ GCLM	[108]
Rat pancreatic cells	10 mM; 4 h	↑ Insulin secretion ↑ HO-1 mRNA and activity	[109]
β-Min6 and HP62 β-cells	100 pM; 2 h	↑ Insulin secretion ↑ cAMP levels ↓ Pde3b, Pde8a and Pde10a mRNA ↓ PDE activity	[110]
INS-1 cells	15 µM; 24 h	↑ Insulin secretion ↑ Insulin mRNA ↑ GLUT2 ↑ Phospho-IRS1, PI3Kp85, AKT protein ↑ PI3K/IRS-1 association ↑ PDX-1 protein ↑ GCK protein	[111]
Wistar rat pancreatic islets	20 µM; 24 h	↑ Cell viability ↑ GLUT2 ↓ Nuclear NF-κB ↑ Phospho-PI3Kp85 ↑ Phospho-Akt ↑ Phospho-GSK3β ↑ Nuclear Nrf-2 ↑ HO-1 ↓ NO production ↓ Cleaved caspase-12, -3, -8 and -9 ↑ Bcl-2 ↓ Bax	[112]
MIN6 cells	10 µM; 24 h	↑ Insulin secretion ↓ Apoptosis ↓ Caspase-3 and caspase-9 activity ↑ Bcl-2/Bax ratio ↓ MDA protein ↑ SOD, catalase, GPx and glutathione activities ↑ Phospho-Akt ↑ Phospho-FoxO1	[113]
INS-1 cells	10:3 weight ratio (22 mg/L); 24 h	↑ Curcumin cellular uptake ↑ Cell viability ↓ Apoptosis ↓ ROS production	[114]
Balb/c mice pancreatic islet cells	12.5 µM; 24 h	↑ Insulin secretion ↑ Glutathione levels ↑ NO production ↓ Apoptosis ↓ Active caspase 3 ↓ Active caspase 9 ↓ Bax protein ↑ Bcl-2 protein	[115]
MIN6 cells	20 µM; 24 h	↑ Cell viability ↓ Ferroptosis ↓ Iron levels ↑ Glutathione levels ↓ GPX4 degradation ↓ Lipid peroxidation	[116]
